# Mechanical energy storage performance of an aluminum fumarate metal–organic framework†
†Electronic supplementary information (ESI) available: Experimental procedures, X-ray diffraction, and molecular simulation. See DOI: 10.1039/c5sc02794b


**DOI:** 10.1039/c5sc02794b

**Published:** 2015-10-05

**Authors:** Pascal G. Yot, Louis Vanduyfhuys, Elsa Alvarez, Julien Rodriguez, Jean-Paul Itié, Paul Fabry, Nathalie Guillou, Thomas Devic, Isabelle Beurroies, Philip L. Llewellyn, Veronique Van Speybroeck, Christian Serre, Guillaume Maurin

**Affiliations:** a Institut Charles Gerhardt Montpellier UMR 5253 CNRS UM ENSCM , Université de Montpellier , CC 15005, Place Eugène Bataillon, F-34095 Montpellier cedex 05 , France . Email: pascal.yot@umontpellier.fr ; Fax: +33 4 67 14 42 90 ; Tel: +33 4 67 14 32 94; b Centre for Molecular Modeling , Ghent University , Technologiepark 903 , B-9052 Zwijnaarde , Belgium; c Institut Lavoisier Versailles , UM 8180 , Université de Versailles St-Quentin , 45, avenue des Etats-Unis , F-78035 , Versailles cedex , France; d PSA Peugeot Citroën – Direction Scientifique et Technologies Futures , DSTF/SEPC/STEP , Route de Gisy – 78943, Velizy-Villacoublay cedex , France; e Aix-Marseille Université , CNRS , MADIREL (UMR 7246) , Centre Scientifique de St. Jérôme , F-13397 , Marseille cedex 20 , France; f Synchrotron Soleil , L'orme des Merisiers , Saint-Aubin – BP 48 , F-91192 Gif-sur-Yvette cedex , France

## Abstract

The aluminum fumarate MOF A520 or MIL-53–FA is revealed to be a promising material for mechanical energy-related applications with performances in terms of work and heat energies which surpass those of any porous solids reported so far. Complementary experimental and computational tools are deployed to finely characterize and understand the pressure-induced structural transition at the origin of these unprecedented levels of performance.

## Abstract 2

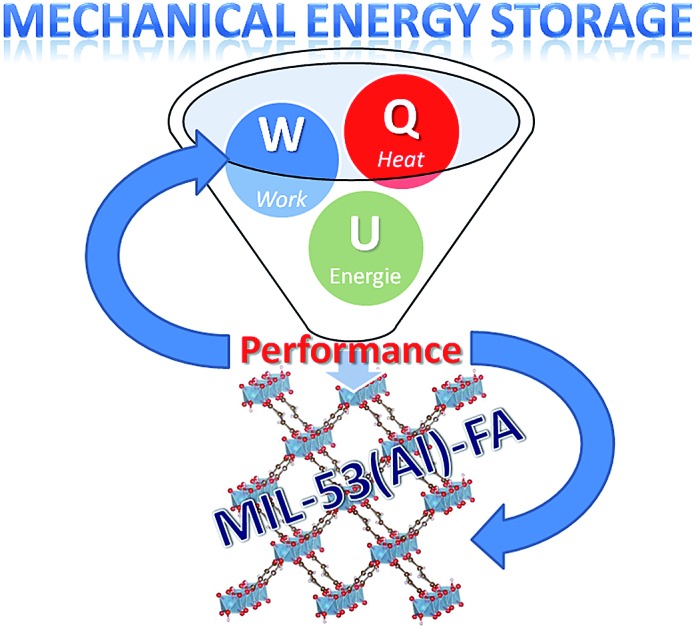
Determination of the mechanical energy storage performance of the aluminum fumarate metal–organic framework A520.

## 1. Introduction

Metal organic frameworks (MOFs) have aroused a great interest over the past decade not only for the wide spectrum of materials that can be synthesized but also for their potential use in societally-relevant applications.^1^ While much effort has been focused on the design of MOFs for gas storage/separation,^1^ much less attention has been paid to tuning their mechanical energy storage performance.^2^–^10^ Indeed, very few hydrophobic MOFs have been reported to absorb relatively high amounts of energy during water intrusion–exclusion cycles.^9^,^10^ Flexible MOFs have been proposed as potential nano-dampers or shock absorbers since their pressure-induced structural transitions in forming a contracted phase can generate relatively high work energy during compression/decompression cycles.^2^–^8^ In particular, Hg-porosimetry and high-pressure X-ray diffraction experiments revealed that the carboxylate-based MIL-53 series^2^,^4^,^7^,^8^ rival or even surpass mesoporous silica and zeolites^9^–^13^ in terms of mechanical energy stored. Very recently, significant improvements have been made to the crystallinity of the commercialized aluminum fumarate A520 14–18 *via* an optimized synthesis route which rendered possible the resolution of the crystal structure of this solid in its hydrated form. This solid, denoted as MIL-53(Al)–FA, was revealed to be isoreticular of the well documented highly flexible MIL-53(Al)–BDC (BDC = 1,4-benzenedicarboxylate) with a slightly smaller pore dimension (7.3 × 7.7 Å^2^*vs.* 8.5 × 8.5 Å^2^),^19^ and interestingly a rigid character upon water sorption. Following the strong shift to higher pressure observed previously for the structural transition when turning from highly flexible MIL-53(Cr, Al) solids to the ‘sorption rigid’ parent MIL-47(V^IV^) analogue,^4^ we assumed here that one could use the Al fumarate features as an attractive candidate to maximize the work energy (*W* = *P* × Δ*V*) absorbed during one compression–decompression cycle through an expected increase in the structural transition pressure (*P*) while maintaining a relatively high volume variation (Δ*V*).^1^

**Fig. 1:**
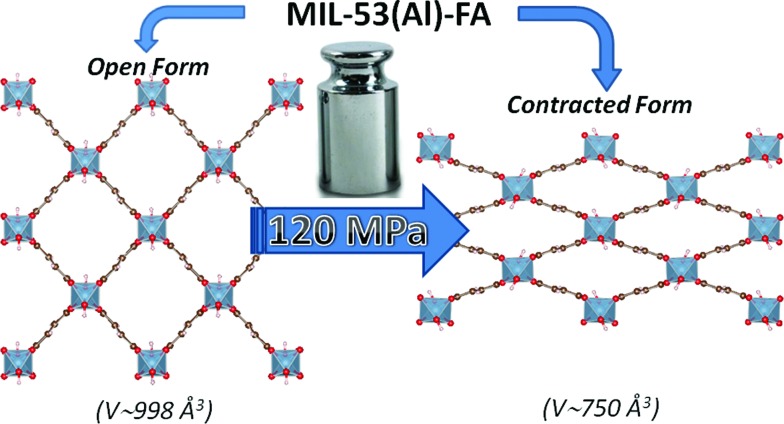
Schematic representation of the pressure-induced contraction of MIL-53(Al)–FA between an open and a contracted form.

Hg-porosimetry and *in situ* high-pressure synchrotron X-ray powder diffraction coupled with molecular simulations confirmed that the dehydrated version of MIL-53(Al)–FA shows a reversible structural contraction (Fig. 1) under an applied pressure above 100 MPa. This leads to a very high work energy of 60 J g^–1^ that considerably exceeds the values reported so far for other porous solids.^2^–^13^ This unprecedented level of performance is maintained with the use of silicon oil, a more environmentally friendly fluid, to perform the compression–decompression cycles. A direct measurement of the heat energy confirms the great promise of this low-cost and stable MOF for such an application.

## 2. Material and methods

Powder of the aluminum fumarate metal–organic framework MIL-53–FA has been prepared following the optimized synthesis route very recently reported by Alvarez *et al.*^18^ The pressure-induced structural response of both the dehydrated and hydrated solids was characterized using mercury intrusion experiments^2^,^4^,^7^ with a porosimeter Micromeritics Autopore 9240. Two intrusion–extrusion (compression–decompression) cycles were applied to the samples in the pressure range 10^–4^ to 420 MPa (see the ESI†). Angle-dispersive X-ray powder diffraction (XRPD) data at high pressure (up to 1.88 GPa) was recorded in-house using filtered Mo-K_α_ (*λ* = 0.710730 Å) and at PSICHE beamline of the Synchrotron Soleil (Saint-Aubin, France) using a monochromatic beam (50 × 50 μm^2^) with a wavelength of *λ* = 0.37380 Å. The pressure was generated with a membrane diamond anvil cell (MDAC) using silicon oil AP 100 (Aldrich) as the pressure-transmitting medium as its kinetic diameter largely exceeds the window size of the fumarate which ensures that it does not enter inside the pores. The applied pressure was determined from the shift of the ruby R1 fluorescence line.^20^ The heat related to the pressure-induced structural transition of the dehydrated solid was determined using a specifically devised calorimetry system^8^ using silicon oil as the pressure-transmitting medium.

Molecular simulations were performed to provide a structural model of the contracted phase detected under applied pressure. This computational effort was based on a new *ab initio* derived flexible force-field for the MOF framework using the QuickFF protocol.^21^ All of the details about the experiments and modelling are available in the ESI.†

## 3. Results and discussion

**Fig. 2:**
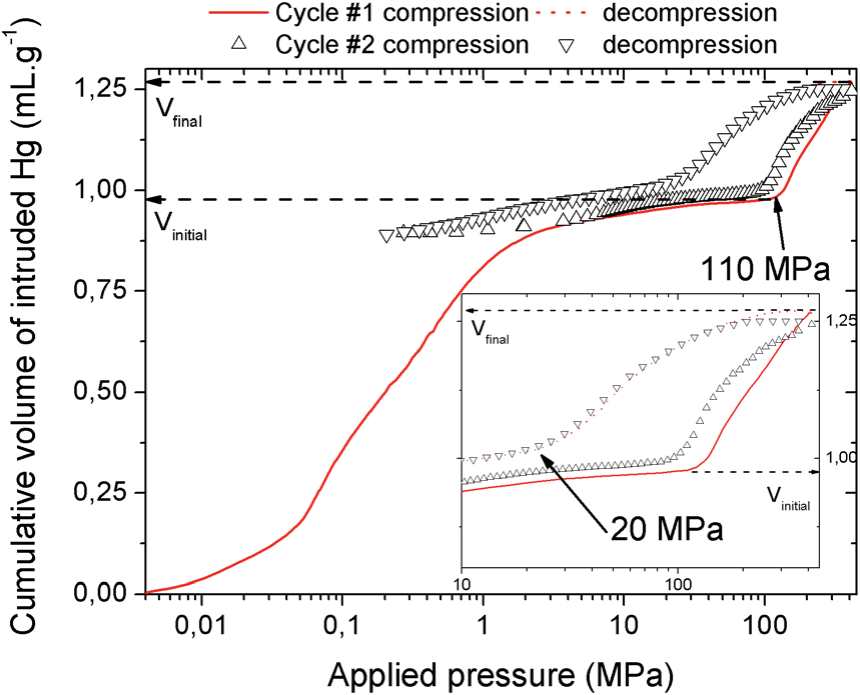
Cumulative volume of intruded mercury in two intrusion–extrusion cycles as a function of the applied pressure obtained for the dehydrated MIL-53(Al)–FA solid (*V*_initial_ and *V*_final_ are the volumes of mercury intruded before and after the contraction of the solid respectively).

The mechanical behavior of the hydrated MIL-53(Al)–FA was first explored through mercury intrusion and angle dispersive XRPD. Fig. S1† reports the evolution of the cumulative volume of intruded mercury as a function of the applied pressure after two intrusion–extrusion (compression–decompression) cycles. Apart from the increase of the volume of Hg intruded below 10 MPa assigned to the compaction of the powder and the filling of the interparticular porosity, this curve does not show any step at higher pressure up to 420 MPa. This observation emphasizes that the hydrated solid does not undergo any structural change in this range of pressure. This also holds true at higher pressure as evidenced by the XRPD patterns collected for this solid which remain unchanged up to 1.88 GPa (Fig. S2†). Referring to our previous computational investigation on the guest-modulation of the mechanical properties of MIL-53(Cr),^22^ the absence of a structural phase transition is not necessarily due to the intrinsic robustness of the MOF framework, but rather to the internal stress exerted by the water molecules which tends to put up resistance to the external applied pressure. To confirm this, the solid was further investigated in its dehydrated form. A structural model was first constructed starting with the crystal structure of the hydrated form and subsequently optimized through Density Functional Theory (DFT) calculations in the absence of the free water molecules (see the ESI†). This led to a structure with the same monoclinic symmetry (SG *P*2_1_/*c*) and a unit cell volume of 985 Å^3^ in good agreement with the experimental value (998.0(1) Å^3^) obtained from XRPD pattern indexing (see Fig. S3†). Mercury porosimetry experiments performed on this solid (Fig. 2) revealed a progressive increase of Hg intruded between 110 MPa and 400 MPa, assigned to a contraction of the structure since Hg cannot penetrate into the micropores.

**Fig. 3:**
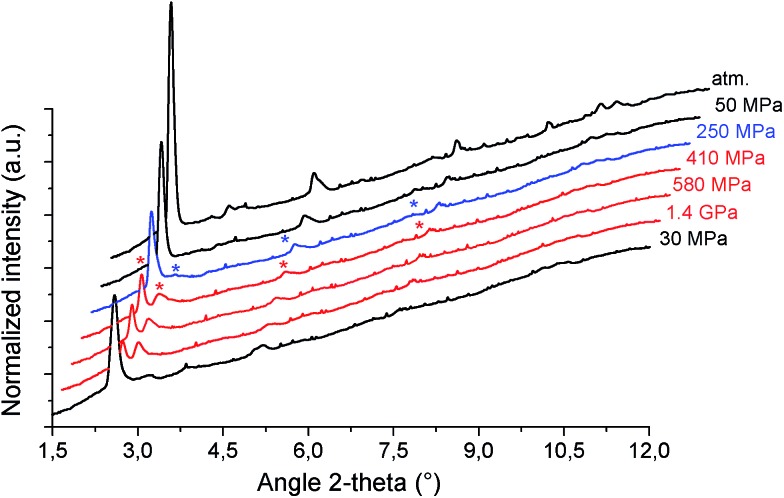
X-ray powder diffraction patterns of MIL-53(Al)–FA as a function of the applied pressure (*λ* = 0.37380 Å). Patterns in black correspond to the pure open form, blue corresponds to a mixture of open and contracted pore forms (* indicates the diffraction peaks assigned to the contracted form) and red corresponds to the contracted form although the presence of a small concentration of the open form is likely to occur.

High pressure XRPD experiments further confirmed a structural change in the same range of pressure with the appearance of new Bragg peaks above 250 MPa (Fig. 3) that are assigned to a more contracted form of MIL-53(Al)–FA. For pressures above 410 MPa the XRPD patterns most probably correspond to the contracted pore form although the presence of a small fraction of the initial structure is likely to occur. The experimental resolution was not of sufficient quality to allow an indexation of the unit cell parameters for the contracted pore structure. It was however possible to estimate the unit cell volume of the contracted phase using the Hg-porosimetry data since we have previously evidenced that the unit cell volume change of the MIL-53 analogues^2^,^4^,^7^,^8^ correlates well with the increase of the volume of intruded Hg. The increase in volume of mercury during the compression step is 0.25 mL g^–1^. Considering a unit cell volume of 998 Å^3^ for the pristine dehydrated structure, this leads to a contracted structure with a unit cell volume of ∼750 Å^3^ which is significantly smaller than the cell dimensions of the pressure-induced phases previously observed for MIL-53(Al)–BDC (820 Å^3^),^7^ MIL-53(Cr)–BDC (931 Å^3^)^23^ and its MIL-47(V) (950 Å^3^)^4^ analogue. Such a larger contraction is due both to the decrease of the cell parameter associated with the presence of a shorter fumarate spacer *vs.* benzyl groups for MIL-53, as well as the resulting absence of π–π interactions.

A computational effort has been further deployed to propose a structural model for this contracted phase. Based on a new *ab initio* derived flexible force-field for the MOF framework (see the ESI†), the energy profile of the MIL-53(Al)–FA structure as a function of its unit cell volume was calculated at 0 K (Fig. S6†). The optimized geometry at a fixed volume of 750 Å^3^ encountered during this energy scan was proposed as a plausible structural model for this contracted phase. The consistency obtained between the theoretical XRPD pattern calculated for this predicted structure and the corresponding experimental data collected at 410 MPa (Fig. S4†) confirmed that the appearance of the new Bragg peaks is due to a contraction of MIL-53(Al)–FA and that the proposed structural model is reliable. In a similar way to the MIL-53–BDC analogues, the structural contraction leads to a significant decrease of the Al–Oc–Cc–Cg2 dihedral angle from 180° (pristine phase) to 155° (contracted phase). This emphasizes that the rotation of the linker about the Oc–Oc axis is also the driving force for the structural transition of MIL-53(Al)–FA.^4^,^7^,^24^

The compression step occurs at a pressure which is significantly higher than that observed either for MIL-53(Al)–BDC (55 MPa), MIL-53(Cr)–BDC (55 MPa), or MIL-47(V)–BDC (85 MPa). This implies that the work energy stored by MIL-53(Al)–FA, that can be calculated from the pressure transition and the corresponding volume variation, attains 60 J g^–1^. This value largely exceeds the performance of the Al–BDC analogue by one order of magnitude and makes MIL-53(Al)–FA the best porous solid reported so far for such an application (see Table 1).

**Table 1:** Comparison of the work energy performance of MIL-53(Al)–FA with that of other porous solids

	Work (J g^–1^)	*P*_transition_ (MPa)	Reference
MIL-53(Al)–FA	60	110	This work
MIL-53(Al)–BDC	7	18	7
MIL-53(Cr)–BDC	16	55	2
MIL-47(V)–BDC	33	125	4
ZIF-8	13.3	—	10
Silicalite	11	—	25
SBA-15 mesoporous silica	4.3–6.1	—	26

**Fig. 4:**
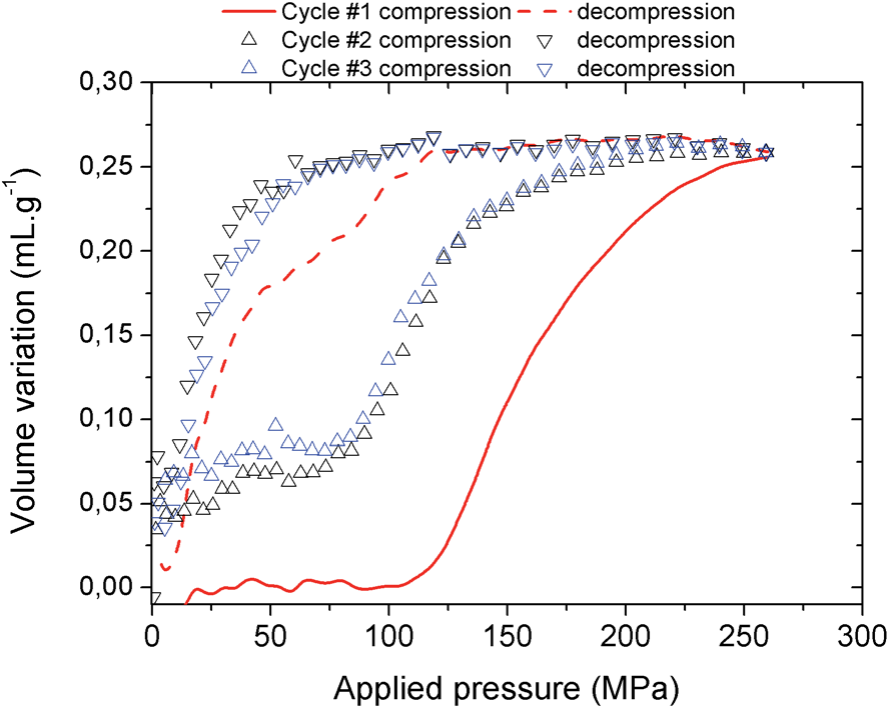
Volume variation of MIL-53(Al)–FA as a function of the applied oil pressure during three compression–decompression cycles.

It is noteworthy that, unlike for MIL-53(Al) where the transition was found to be irreversible, mercury intrusion experiments further evidenced that MIL-53(Al)–FA shows a fully reversible mechanical behavior upon compression–decompression cycles with the presence of a hysteresis of about 125 MPa. This was confirmed using high pressure XRPD which revealed that the contracted version of MIL-53(Al)–FA returns to the initial form once the pressure is released (Fig. 3). In conjunction with its industrial availability (A520), these observations make this solid an exceptional candidate for mechanical energy storage applications and particularly in the form of nano-dampers. However for the purposes of application, mercury cannot be considered as a pressure transmitting medium due to its very high toxicity. We envisaged as a further step the use of a more environmentally friendly fluid, using silicon oil to perform cycles of compression/decompression on MIL-53(Al)–FA (see the ESI†). The corresponding data are reported in Fig. 4.

In contrast with the Hg experiment, the increase of the volume at low pressure is not present anymore as the silicone oil is a wetting fluid that can spontaneously occupy the interparticular porosity. A step for cycle 1 occurs in the pressure range 100–250 MPa and leads to a volume variation of 0.25 mL g^–1^. Both observations concur very well with the values obtained with mercury porosimetry, the lower upper pressure *vs.* Hg being associated with the limit of the current oil system (250 MPa) compared to the mercury set-up (400 MPa). This strongly supports that the selected silicon oil is bulky enough not to penetrate into the MOF micropores and hence this fluid can be used to allow the monitoring of the pressure-induced structural transition of MIL-53(Al)–FA.

The silicon oil compression–decompression cycle presents a hysteresis which is consistent with the Hg porosimetry and the work energy stored, 41.7 J g^–1^, remains very high. Both features confirm the promise of this solid as a potential nano-damper. A partial loss of volume and a decrease of the transition pressure were recorded after the first compression (from 0.25 mL g^–1^ and 100–250 MPa for the first cycle to 0.20 mL g^–1^ and 72–250 MPa for the other cycles respectively) which might be due to the presence of silicon oil at the pore aperture of the MOF at the outer surface of the particles.^8^ However, the performance in terms of the work energy stored remains very high (22.9 J g^–1^, see Table 2) and the cycles are superimposable, within experimental error.

**Table 2:** Experimental energetic data of compression/decompression cycles on the MIL-53(Al)–FA

	Work (J g^–1^)	Heat (J g^–1^)	Internal energy (J g^–1^)
Cycle 1: compression	41.7	–25.1	16.6
Cycle 1: decompression	–10.8	6.4	–4.4
Cycle 2: compression	22.9	–18.7	4.2
Cycle 2: decompression	–8.0	6.3	–1.7
Cycle 3: compression	22.2	–18.2	4.0
Cycle 3: decompression	–8.8	6.5	–2.3

**Fig. 5:**
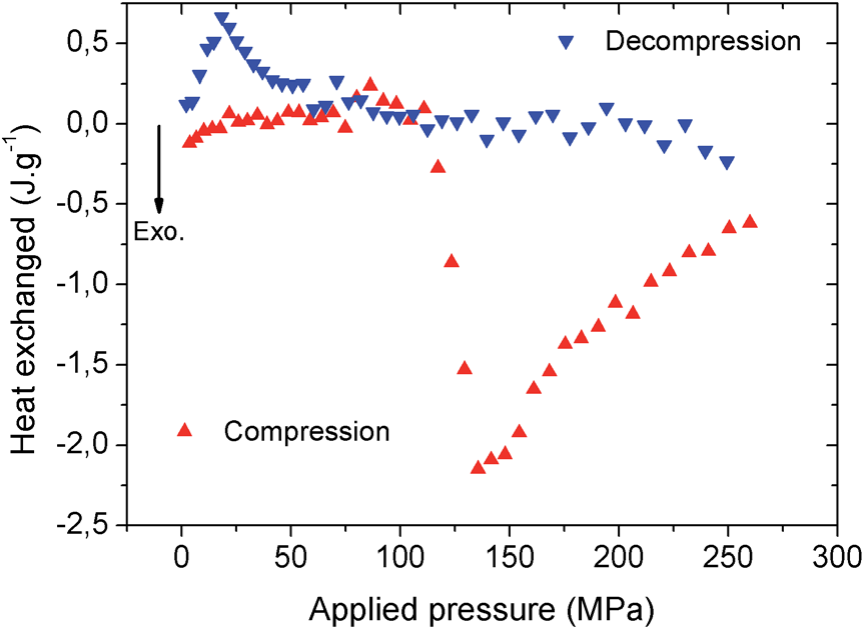
Heat energy obtained for MIL-53(Al)–FA as a function of the pressure during the first cycle release. Red upwards-pointing triangles correspond to compression and blue downwards-pointing triangles correspond to decompression.

The heat dissipated by the structural transition of MIL-53(Al)–FA during the first compression/decompression cycle was further assessed using calorimetry measurements. The corresponding data are reported in Fig. 5.

It is shown that the compression (contraction of the structure) is exothermic while the decompression (expansion of the structure) is endothermic and this trend is consistent with that previously reported for MIL-53(Al)–BDC.^8^ Table S1† evidences that in terms of dissipated heat energy, MIL-53(Al)–FA also largely outperforms all of the porous solids, *i.e.* other MOFs and hydrophobic silica. It is also shown that after the first cycle, the heating energy (*i.e.* the difference between the heat of compression and decompression energy) is around –18.7 J g^–1^ which is significantly higher than the value obtained for MIL-53(Al)–BDC (–5 to –6 J g^–1^ during cycle 1).^8^

This suggests that a heat evacuation protocol would need to be implemented for the use of this solid as a nano-damper. Finally, Table 2 emphasizes that the work and heat energies are significantly different resulting in internal energy (*U*) values (–8.4 to 6.9 J g^–1^) which are much higher than the value previously reported for MIL-53(Al)–BDC (–3.0 to 1.0 J g^–1^).

## 4. Conclusions

The aluminum fumarate MIL-53–FA or A520 represents the best porous solid reported so far for mechanical-energy related applications, by virtue of its reversible structural switching to form a more contracted phase that can be provoked by the application of a high external pressure, resulting in outstanding performances in terms of work and heat energies. This commercialized material is particularly attractive since its low-production cost, low toxicity and high stability will not be a drawback for further device development following the concept of a MOF/silicon oil system proposed in this study.

## Supplementary Material

Supplementary information
