# Supplementary material for: Mechanical energy storage performance of an aluminum fumarate metal–organic framework
Source: Chem Sci. 2015 Oct 5;7(1):446–50. doi: 10.1039/c5sc02794b (PMC5952545; doi:10.1039/c5sc02794b)
Supplement: Supplementary file 1 [file SC-007-C5SC02794B-s001.pdf]

Electronic Supplementary Material (ESI) for Chemical Science.
This journal is © The Royal Society of Chemistry 2015

Electronic Supporting Information

Mechanical energy storage performances of the Aluminum Fumarate Metal–Organic Framework A520

Pascal. G. Yot,[*a] Louis Vanduyfhuys,[b] Elsa Alvarez,[c] Julien Rodriguez,[d] Jean-Paul Itié,[e] Paul Fabry,[c] Nathalie Guillou,[c] Thomas Devic,[c] Isabelle Beurroies,[d] Philip L. Llewellyn,[d] Veronique Van Speybroeck,[b] Christian Serre,[c] Guillaume Maurin,[a]

a. Institut Charles Gerhardt Montpellier (UMR 5253), Université de Montpellier, CC 15005, Place Eugène Bataillon, F-34095 Montpellier cedex 05, France.
e-mail: pascal.yot@umontpellier.fr; Tel: +33 4 67 14 32 94; Fax: +33 4 67 14 42 90.

b. Centre for Molecular Modeling, Ghent University, Technologiepark 903, B-9052 Zwijnaarde, Belgium. Centre for Molecular Modeling, Ghent University, Technologiepark 903, B-9052 Zwijnaarde, Belgium.

c. Institut Lavoisier Versailles (UMR 8180), Université de Versailles St-Quentin, 45, avenue des Etats-Unis, F-78035 Versailles cedex, France.

d. Aix-Marseille Université, CNRS, MADIREL (UMR 7246), Centre Scientifique de St. Jérôme, F-13397 Marseille cedex 20, France.

1. Investigation of the hydrated MIL-53(Al)-FA.

Considering the pore size of MOFs materials (and pressure range explored by mercury intrusion ($P \leq 420$ MPa)) the non-wetting mercury cannot penetrate into the pores, hence the pressure increase induces an isostatic pressure on the crystallites. The volume of intruded mercury is then directly related to the volumetric strain corresponding to the compressibility of the material. The hydrostatic compression experiments were performed using a Hg porosimeter Micromeritics Autopore 9240 on outgassed (~6.5 Pa, 1 h) powdered MOF samples. The pressure applied can vary from 0.1 to 420 MPa. In the explored range of pressure, using the Washburn's law $P = -\frac{4\gamma\cos\theta}{d}$ with γ mercury surface tension and θ the contact angle of 0.485 N/m and 130° respectively, the pore diameters accessible for Hg would range in the interval [2×10^{7}–400 Å].

1.1. Mercury intrusion

The sample was preliminary outgassed at room temperature for 1 hour and then loaded into the penetrometer under air atmosphere. The two steps present in the mercury intrusion

curve below 10 MPa correspond to the filling of the penetrometer, the interparticle porosity and of the textural effects. No pressure induced compression of the porous solid is detected.

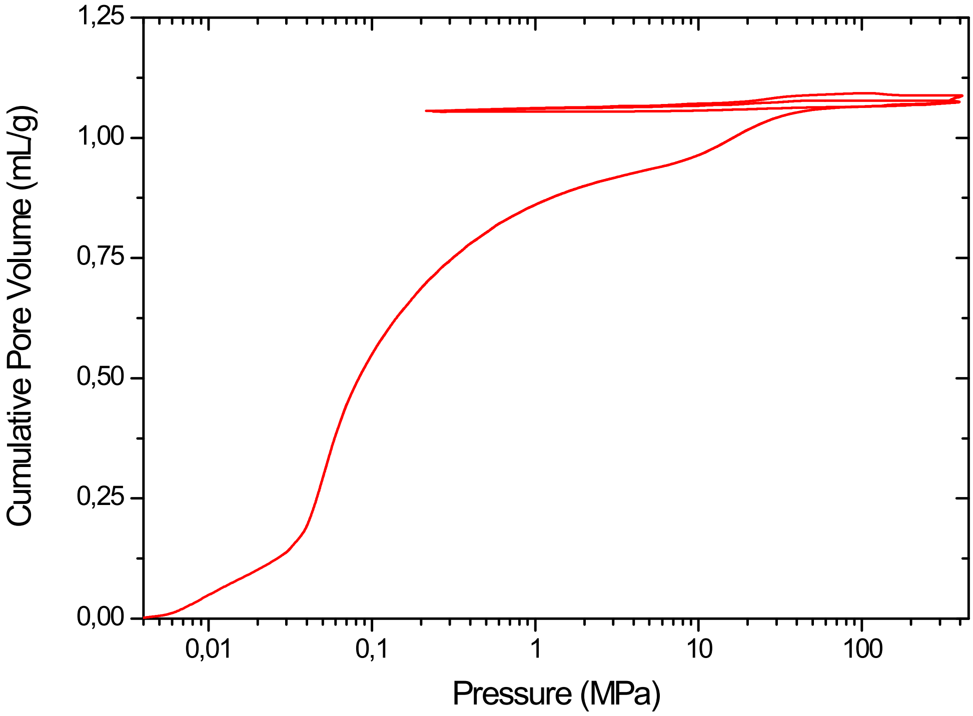

Figure S1. *Cumulative volume of intruded mercury in a two cycles Intrusion–extrusion as a function of the applied pressure obtained for the hydrated Aluminum Fumarate Metal–Organic Framework A520.*

1.2. Angle dispersive X-ray diffraction

Angle-dispersive X-ray diffraction data at high pressure was performed in-house using filtered Mo-Kα (λ=0.710730 Å). The pressure was generated with a Merill-Basset diamond anvil cell and was determined from the shift of the ruby R1 fluorescence line [1]. Silicon oil AP 100 from Aldrich was used as pressure transmitting medium to generate hydrostatic conditions into the pressure cell.

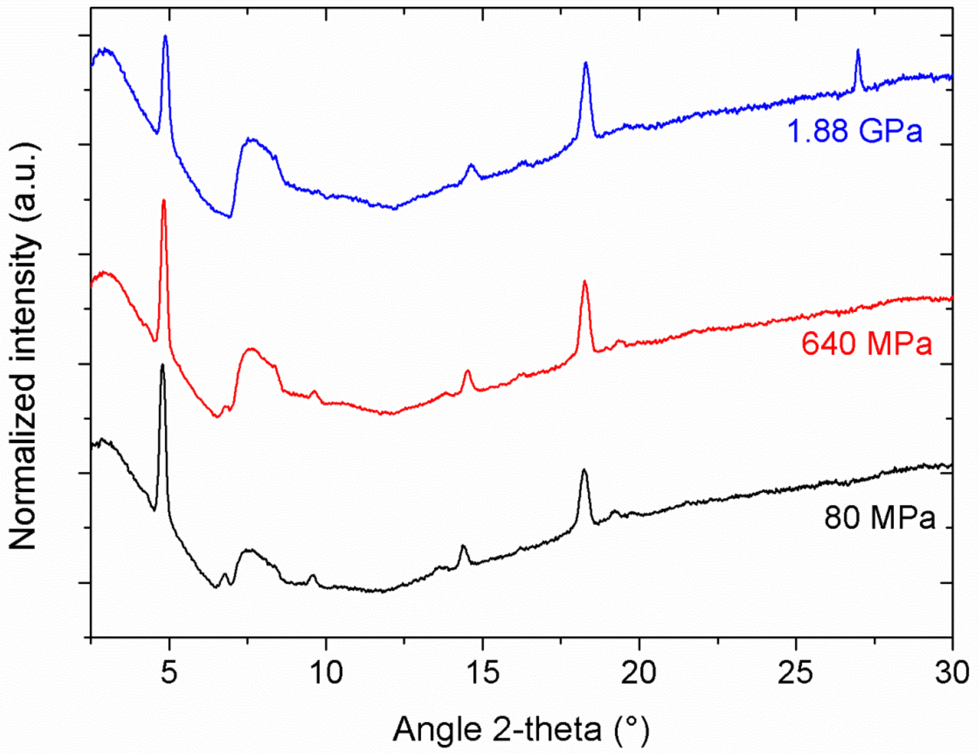

Figure S2. *Sequential diffraction patterns obtained on MIL-53(Al)-FA in the pressure range 0.80-2 GPa.*

2. Investigation of the dehydrated MIL-53(Al)-FA.

2.1. Mercury intrusion

The solid was previously activated at 110°C under secondary vacuum during 8 hours and loaded into the penetrometer for the mercury intrusion under dry argon atmosphere into a glove box (H_2O<1ppm) to avoid rehydratation. Two compression-decompression cycles were applied on the sample in the range 10^{-4}-420 MPa using the same Hg-porosimeter than mentioned above.

2.2. Powder X-ray diffraction

The dehydrated form of MIL-53(Al)-FA was preliminary studied in-house. The powder was loaded into glass capillaries of 0.5 mm diameter. The dehydration was performed at 110°C during 8 hours under secondary vacuum and sealed to avoid rehydration. Powder X-ray diffraction data were collected using a PANalytical X'PERT II diffractometer using a monochromatic Cu K_α source (λ = 1.5406 Å) with an operating voltage of 40 kV and a beam current of 40 mA. The patterns were collected for 2θ = 5−70°. The unit-cell parameters of

the dehydrated form of MIL-53(Al)-FA were determined by indexing and refining the X-ray powder patterns, using JANA 2006 suite [2].

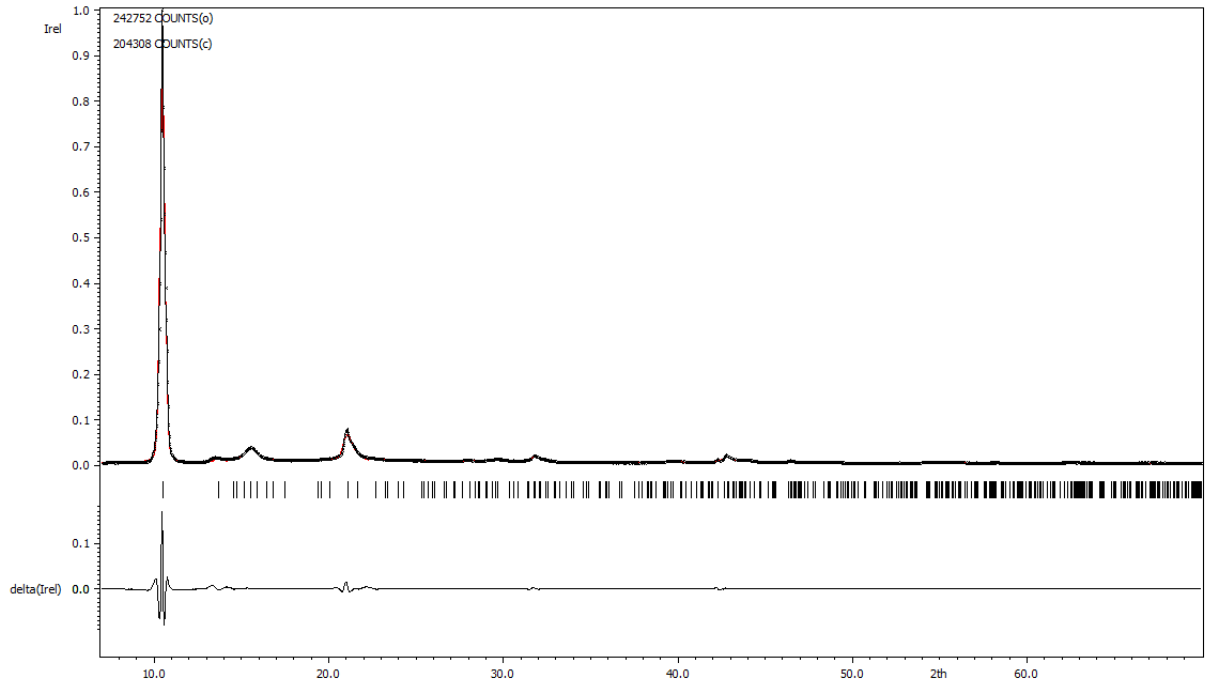

Figure S3. *Structure-independent refinement of the unit-cell of the diffraction pattern obtained for the dehydrated MIL-53(Al)-FA, space group $P2_1/c$: a=7.022(3) Å, b=12.154(3) Å, c=14.745(5) Å, β=127.62(2)°, V=998.0(1)$Å^3$ (Rp: 7.81, Rwp: 10.66).*

In situ powder X-ray diffraction was carried out on PSICHE beamline at the French national synchrotron source (Synchrotron Soleil, Saint-Aubin, France) using a membrane diamond anvil cell (MDAC) to generate the external pressure. The patterns were collected at room temperature using a monochromatic beam (50×50 μm^2) with the wavelength of λ=0.37380 Å. and a Mar detector. The sample distance-detector, parameters of the detector and wavelength were calibrated using NIST standard LaB_6. All the two dimensional images were integrated using Fit2D software [3] .The sample was previously dehydrated in the same conditions (100°C, 8 hours under secondary vacuum) and loaded into a glove bag on the beam line. Hydrostatic conditions were ensured by the addition of silicon oil (AP 100, Aldrich) as pressure transmitting media. During the experiment the pressure generated by the MDAC and was determined from the shift of the ruby R1 fluorescence line [1].

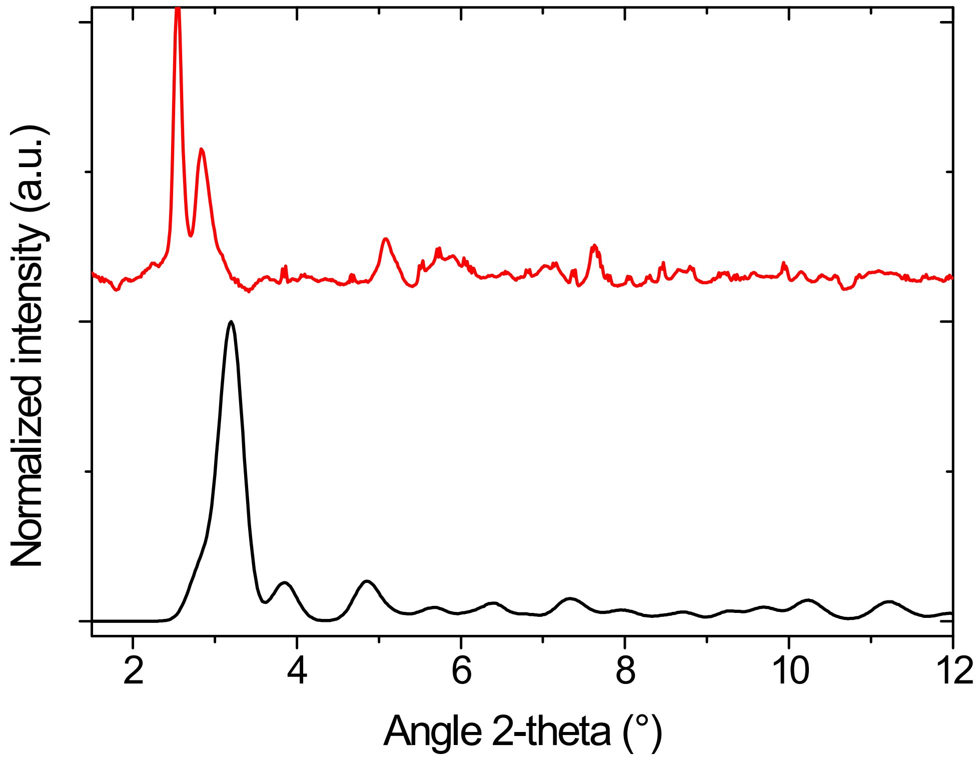

Figure S4. *Experimental XRPD pattern obtained for MIL-53(Al)-FA under 410 MPa (red) and XRPD pattern calculated from the predicted structure model for the contracted phase (black) under 410 MPa (λ=0.3738 Å).*

2.3. Computational details

2.3.1. Structure solution of the dehydrated form of MIL-53(Al)-FA

A structure model for the dehydrated MIL-53(Al)-FA was built starting with the crystal structure previously elucidated for the hydrated solid [4] and subsequent DFT geometry optimization was performed with the Quickstep module available as part of the CP2K code [5]. In these simulations both the positions of the atoms of the framework and the cell dimensions were fully relaxed. The PBE functional [6] was used along with a combined Gaussian basis set and planewave pseudopotential strategy as implemented in the code. A triple zeta basis set (TZVP-MOLOPT) was considered for all atoms, except for the Al centres, where double zeta functions were employed (DZVP-MOLOPT) [7]. The pseudopotentials used for all of the atoms were those derived by Goedecker, Teter and Hutter [8]. The van der

Waals effects interactions were taken into account via the use of the semi-empirical dispersion corrections as implemented in the DFT-D3 method [9].

2.3.2. Force Field derivation for the flexible MIL-53(Al)-FA framework

An *ab initio* derived force field was constructed for the MIL-53(Al)-FA framework using the general QuickFF protocol [10]. The *ab initio* data needed for the FF derivation consist of the geometry and Hessian in equilibrium. These input data were generated for the cluster as shown in Figure S6. This cluster represents a diamond shaped unit, terminated with water molecules and hydroxyl groups. This termination was chosen such that the total charge is minimal (+4 in this case) and the spin is zero.

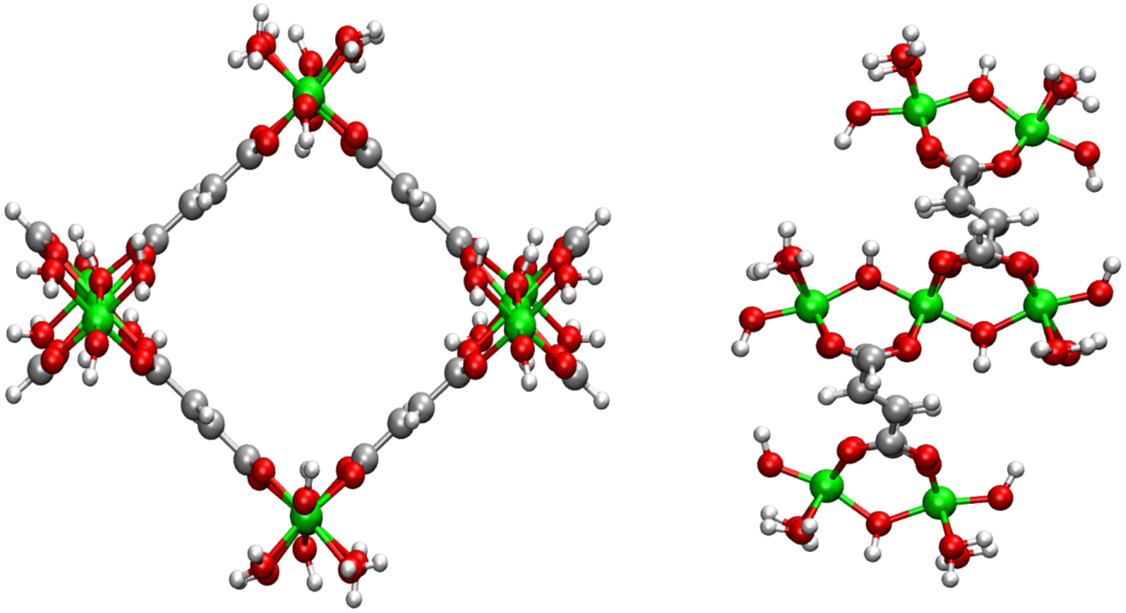

Figure S5. *top view (left) and side view (right) of the isolated cluster used to derive a force field.*

The geometry was first optimized in order to compute the equilibrium structure. Next, the Hessian was calculated in the equilibrium configuration. Both computations were performed using the Gaussian 09 [11] package with the B3LYP [11-12-13-14] functional, the cc-pVTZ basis set and the scf=qc and int(grid=ultrafine) options. The potential energy of the force field has the same expression as in QuickFF:

$$E = \sum_{i=1}^{N_r} \frac{K_i^r}{2}(r_i - r_{0,i})^2 + \sum_{j=1}^{N_\theta} \frac{K_j^\theta}{2}(\theta_j - \theta_{0,j})^2$$

$$+ \sum_{k=1}^{N_\psi} \frac{K_k^\psi}{2}\left[1 - cos\left(m_k(\psi_k - \psi_{0,k})\right)\right] + \sum_{l=1}^{N_d} \frac{K_l^d}{2}(d_l - d_{0,l})^2$$

$$+\frac{1}{2}\sum_{i,j=1}^{N_{at}}\frac{q_i q_j}{r_{ij}}erf\left(\frac{r_{ij}}{d_{ij}}\right)+\frac{1}{2}\sum_{i,j=1}^{N_{at}}\varepsilon_{ij}\left[1.84\cdot 10^5\cdot exp\left(-\frac{r_{ij}}{\sigma_{ij}}\right)-2.25\left(\frac{\sigma_{ij}}{r_{ij}}\right)^6\right]$$

In the expression for the electrostatic interactions, Gaussian charge distributions are used and all pairs are included, i.e. no exclusion rules are taken into account, while for the van der Waals interactions, 1-2, 1-3 and 1-4 pairs are excluded. This approach was successfully followed earlier in the derivation of the force field for the MIL-53(Al) framework [15]. First, the atomic charges are derived from the molecular electron density of the equilibrium structure using a recently developed charge scheme namely the Minimal Basis Iterative Stockholder scheme [16]. Next, QuickFF was applied to construct the covalent part of the force field. Finally, the van der Waals interactions of the MM3 force field of Allinger et al. [17] were added a posteriori, with a global scaling factor of 0.85 applied to all ε_{ij} parameters and a global scaling factor of 1.20 applied to all σ_{ij} parameters. The ε-scaling was taken from a previous study on MIL-53 with the BDC linker [18], while the σ-scaling was applied to result in a better agreement between FF prediction and experimental measurement of the closed pore geometry obtained after applying pressure.

Using this newly developed force field, the geometry of the MIL-53(Al)-FA was optimized to find the open pore equilibrium structure. Furthermore, an energy scan was performed with the force field to compute the energy at 0 K as function of unit cell volume. A series of geometric optimizations were performed in which the interdiagonal angle θ, i.e. the angle between the *b* and *c* unit cell vectors, was constrained to a certain value between 40° and 140°, whereas all nuclear coordinates and other unit cell degrees of freedom were relaxed. The minimal energy was computed for each value of θ and the resulting energy profile is shown in Figure S7 as a function of the unit cell volume corresponding to each value of θ.

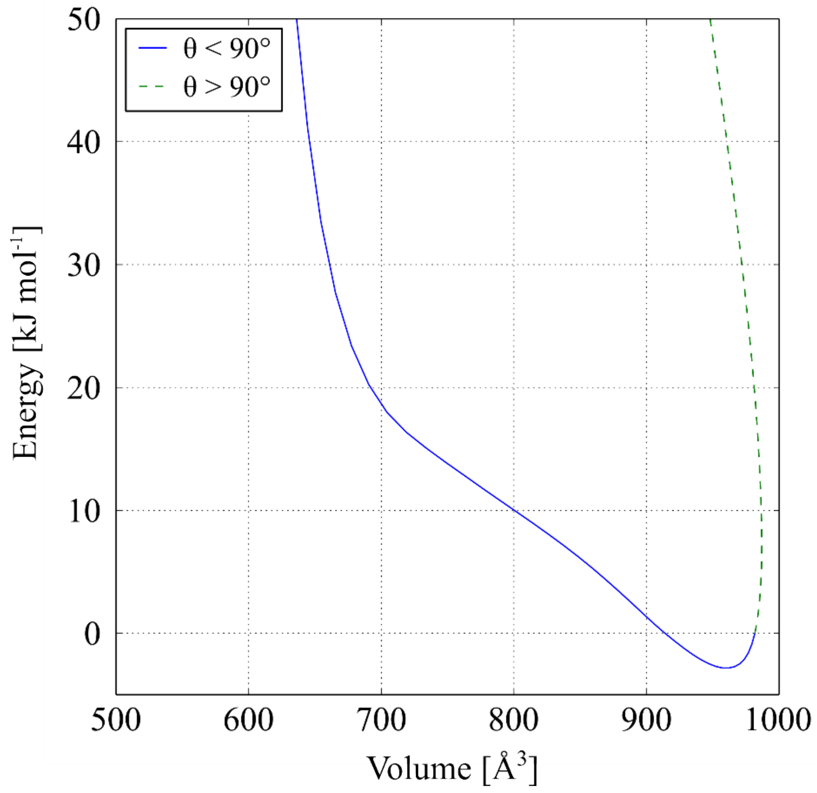

Figure S6. *Energy (at 0 K) of MIL-53(Al)-FA as a function of the volume computed by means of a series of constrained geometry optimizations. The blue solid curve represents structures with an interdiagonal angle smaller than 90° (i.e. b > c), while the green dashed line represents structures with an interdiagonal angle larger than 90° (i.e. b < c).*

The energy profile clearly reveals that no stable narrow pore minimum exists at 0K. This indicates that the system will only remain in a closed pore structure if a pressure is applied and maintained. Once the pressure is relieved, the system will relax back to the large pore structure. This explains why the pressure-induced transition of MIL-53(Al)-FA is reversible, in contrast to the original MIL-53(Al) with BDC linkers.

2.4. Determination of work and heat energies of the structural transition

The sample is weighted in a high pressure cell and heated under vacuum (2.10^{-4} mbar) to 200°C during 16 hours in order to evacuate residual adsorbed molecules such as water. The evacuated cell is then placed in the microcalorimeter and connected to the syringe pump as reported in [19]. The system is further evacuated under a primary pump. After thermal equilibrium, the syringe pushes the silicone oil (AP 100, Aldrich) until filling the cell to a pressure of 2.0 MPa. The pressure is then increased stepwise up to 250 MPa by injection of small volumes of oil in the system every hour (30 µL at each step). The sample volume, the

equilibrium pressure and the heat evolved are measured at each step. A blank experiment is carried out without sample to subtract from the raw data the variations of volume of oil due to its compressibility, as well as its heat of compression/decompression.

The oil compressive system allows the full thermodynamic analysis of the data. One can then consider the internal energy (*U*) of the system. The heat (*Q*) and work (*W*) produced during the compression/decompression. Applying the first principle where the change in internal energy (*U*) is given by $\Delta U = Q + W$, it is possible to calculate this transition energy.

The work *W* provided to the system is obtained from the $P = f(V)$ data by integration between points A and B, after subtraction of the oil compressibility:

$$W = -\int_A^B PdV \quad (1)$$

The heat produced between A and B is calculated from the sum of heats at each pressure step after subtraction of the oil compressibility. The experimentally measured heat (Q_{exp}) is the sum of the heat of compressibility for the MOF (Q_W) and for the silicone oil (Q_{oil}).

$$Q_{exp} = Q_W + Q_{oil} \quad (2)$$

The heat of compressibility for the silicone oil can be calculated using the Eq. 2, to subtract the oil contribution of the experimental measured heat.

For the mercury intrusion experiment shown in figure S8, the sample is treated at 200°C under vacuum and then sealed in a glove box under argon with grease such that the contact with air is negligible. Then the experiment is carried out by first filling the cell to 6.6 Pa and then mercury is introduced step by step up to 400 MPa.

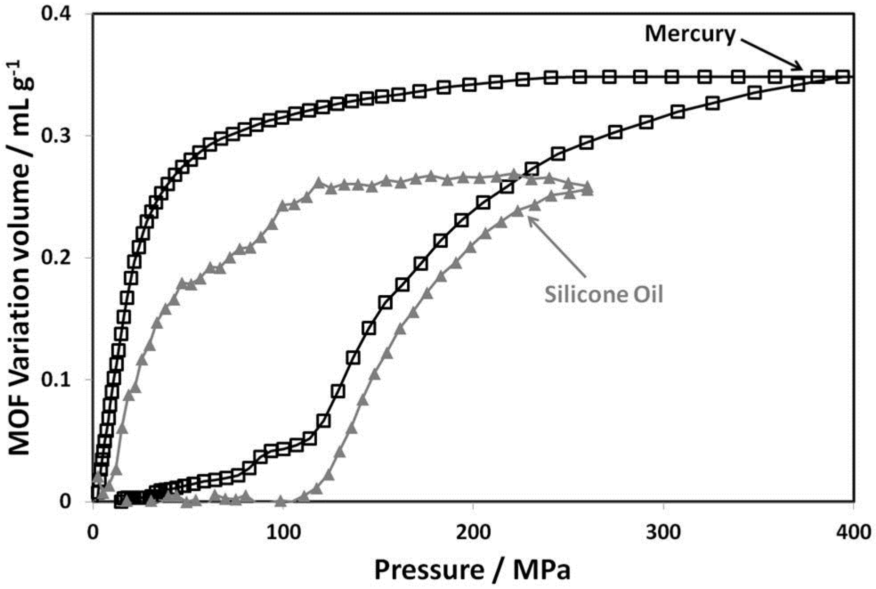

Figure S8. *Aluminium Fumarate MOF volume variation as a function of the applied pressure of mercury and oil during the first cycle.*

The oil system is currently limited to 250 MPa, and it is evident from Figure S8 that the Aluminium Fumarate compression is not complete. Indeed, the comparison with mercury intrusion suggests that the Aluminium Fumarate compression continues at least up to 400 MPa. It thus follows that the work and heat energies measured with this sample in the oil compressive system, limited to 250 MPa, may be underestimated.

	P_{comp} (MPa)	P_{dec} (MPa)	Volume variation ($mL.g^{-1}$)	W_{stored} ($J.g^{-1}$)	W_{diss} ($J.g^{-1}$)	Ref.
**Compression-decompression**						
MIL-53(Al)*	33	7	0.23	7.7	5.9	[19]
MIL-53(Al)**	15	0	0.24	6.6	6.6	[18]
MIL-47(V)**	85	60	0.3	33	10	[20]
MIL-53(Cr)**	55	10	0.25	13.75	11.25	[21]
**Water intrusion-extrusion**						
ZIF-8	27	22	0.5	13.3	2.1	[22]
Silicalite	96	91	0.11	11	0.7	[23]
Pure silica ITQ-12 (ITW) Zeolite	172	172	0.05	8.1	0	[24]
Pure silica ITQ-4 (ITW) zeolite	42	0	0.14	5.7	5.7	[25]

* Compression with silicon oil ** Compression with mercury

Table S1. *Characteristic of compression-decompression and water intrusion-extrusion for different matrices.*

REFERENCES

[1] H. K. Mao, J. Xu, P. M. Bell, *J. Geophys. Res.*, 1986, **91**, 4673.

[2] V. Petricek, M. Dusek, L. Palatinus, *Z. Kristallogr.*, 2014, **229**, 345.

[3] A. P. Hammersley, S. O. Svensson, M. Hanfland, A. N. Fitch, D. Häusermann, *High Press. Res.*, 1996, **14**, 235.

[4] E. Alvarez, N. Guillou, C. Martineau, B. Bueken, B. Van de Voorde, C. Le Guillouzer, P. Fabry, F. Nouar, F. Taulelle, D. de Vos, J.-S. Chang, K. Ho Cho, N. Ramsahye, T. Devic, M. Daturi, G. Maurin, C. Serre, *Angew. Chem. Int. Ed.*, 2015, **54**, 3664.

[5] J. VandeVondele, M. Krack, F. Mohamed, M. Parrinello, T.Chassaing, J. Hutter, *Comput. Phys. Commun.*, 2005, **167**, 103.

[6] J. P. Perdew, K. Burke, M. Ernzerhof, *Phys. Rev. Lett.*, 1996, **77**, 3865.

[7] J. VandeVondele, J. Hutter, *J. Chem. Phys.*, 2007, **127**, 114105.

[8] S. Goedecker, M. Teter, J. Hutter, *Phys. Rev. B.*, 1996, **54**, 1703-1710.

[9] S. Grimme, J. Antony, S. Ehrlich, H. Krieg, *J. Chem. Phys.*, 2010, **132**, 154104.

[10] L. Vanduyfhuys, S. Vandenbrande, T. Verstraelen, R. Schmid, M. Waroquier, V. Van Speybroeck. *J. Comput. Chem.*, 2015, **36**, 1015.

[11] M. Frisch et al., Gaussian 09 Revision A.02, Gaussian Inc., Wallingford, CT, 2009.

[12] A. Becke, *Phys. Rev. A*, 1988, **38**, 3098.

[13] A. Becke, *J. Chem. Phys.*, 1993, **98**, 5648.

[14] C. Lee, W. Yang, R. Parr, *Phys. Rev. B*, 1988, **37**, 785.

[15] L. Vanduyfhuys, T. Verstraelen, M. Vandichel, M. Waroquier, V. Van Speybroeck, *J. Chem. Theory Comput.*, 2012, **8**, 3217.

[16] T. Verstraelen et al., in preparation; T. Verstraelen, K. Boguslawski, P. Tecmer, F. Heidar-Zadeh, M. Chan, T. D. Kim, Y. Zhao, S. Vandenbrande, D. Yang, C. E. González-Espinoza, P. A. Limacher, D. Berrocal, A. Malek, P.W. Ayers HORTON 2.0.0, http://theochem.github.com/horton/, 2015.

[17] N. Allinger, Y. Yuh, J. Lii, *J. Am. Chem. Soc.*, 1989, 111, 8551.

[18] P.G. Yot, Z. Boudene, J. Macia, D. Granier, L. Vanduyfhuys, T. Verstraelen, V. Van Speybroeck, T. Devic, C. Serre, G. Férey, N. Stock, G. Maurin, *Chem. Comm.*, 2014, **50**, 9462.

[19] J. Rodriguez, I. Beurroies, T. Loiseau, R. Denoyel, P. L. Llewellyn, *Angew. Chem. Int. Ed.*, 2015, **54**, 4626.

[20] P.G. Yot, Q. Ma, J. Haines, Q. Yang, A. Ghoufi, T. Devic, C. Serre, V. Dmitriev, G. Férey, C. Zhong, G. Maurin, *Chem. Sci.*, 2012, **3**, 1100.

[21] I. Beurroies, M. Boulhout, P. L. Llewellyn, B. Kuchta, G. Férey, C. Serre, R. Denoyel, *Angew. Chem., Int. Ed.*, 2010, **49**, 7526.

[22] G. Ortiz, H. Nouali, C. Marichal, G. Chaplais, J. Patarin, *Phys.Chem. Chem. Phys.*, 2013, **15**, 4888.

[23] L. Tzanis, H. Nouali, T. J. Daou, M. Soulard, J. Patarin, *Mater. Lett.*, 2014, **115**, 229.

[24] I. Khay, L. Tzanis, T. J. Daou, H. Nouali, A. Ryzhikov, J. Patarin, *Phys. Chem. Chem. Phys.*, 2013, **15**, 20320.

[25] M. A. Saada, S. Rigolet, J.-L. Paillaud, N. Bats, M. Soulard, J. Patarin, *J. Phys. Chem. C*, 2010, **114**, 11650.
